# Against Moral Responsibilisation of Health: Prudential Responsibility and Health Promotion

**DOI:** 10.1093/phe/phz006

**Published:** 2019-05-25

**Authors:** Rebecca C H Brown, Hannah Maslen, Julian Savulescu

**Affiliations:** Oxford Uehiro Centre for Practical Ethics, University of Oxford

## Abstract

In this article, we outline a novel approach to understanding the role of responsibility in health promotion. Efforts to tackle chronic disease have led to an emphasis on personal responsibility and the identification of ways in which people can ‘take responsibility’ for their health by avoiding risk factors such as smoking and over-eating. We argue that the extent to which agents can be considered responsible for their health-related behaviour is limited, and as such, state health promotion which assumes certain forms of moral responsibility should (in general) be avoided. This indicates that some approaches to health promotion ought not to be employed. We suggest, however, that another form of responsibility might be more appropriately identified. This is based on the claim that agents (in general) have prudential reasons to maintain their health, in order to pursue those things which make their lives go well—i.e. that maintenance of a certain level of health is (all-things-considered) rational for many agents, given their pleasures and plans. On this basis, we propose that agents have a self-regarding *prudential* responsibility to maintain their health. We outline the implications of a prudential responsibility approach to health promotion.

## Introduction

This article addresses the legitimacy of so-called ‘responsibilising’ approaches to state health promotion. Typically aimed at encouraging people to adopt healthy lifestyles, such approaches sometimes instruct agents to ‘take responsibility’ for their health, or communicate that agents are morally responsible—and potentially subject to moral criticism—for their (poor) health. We interrogate the claim that agents are, and should be, considered responsible for their health, and the extent to which moral responsibility is relevant to assessments of health-related behaviour.

This article comprises of two parts. In Part I, we discuss how different approaches to health promotion through behaviour change emphasise the role of individual responsibility, and to what extent such allocations of responsibility are philosophically coherent and ethically appropriate. In Part II, we consider whether the kinds of reasons people have to adopt healthy behaviours ground moral responsibility. We distinguish between moral and prudential reasons, and suggest that people’s reasons to adopt healthy behaviours are primarily prudential in nature, rather than moral. We argue that these prudential reasons ground a self-regarding ‘prudential responsibility’ for many agents to adopt behaviours that maintain a certain level of health, to the extent that this level of health is necessary for maintaining and increasing the agent's well-being. Accordingly, prudential responsibility permits only some limited forms of responsibilising health promotion.

## Part I: Moral Responsibility and Health-Related Behaviour

### Background: Chronic Disease, Health Promotion and Responsibilising Policy

An important focus of health promotion efforts is the reduction of chronic diseases, which are by far the leading cause of death, responsible for 68 per cent of deaths globally (WHO, 2014). Reducing chronic disease has been identified as a priority by numerous national governments and leading healthcare organisations, with World Health Organisation (WHO) member states pledging to reduce premature mortality from four major chronic diseases (heart disease, lung disease, cancer and diabetes) by a third by 2030. Consequently, public health has increased its focus on changing so-called ‘lifestyle’ behaviours in order to reduce people’s exposure to risk factors, such as unhealthy diets, lack of physical activity, tobacco smoke and excessive alcohol consumption.

Notions of responsibility are built into policies and practices of healthcare in varying ways. It is, however, often unclear *what kind* of responsibility is being used, and whether the inclusion of responsibility in the framing of both the ‘problem’ (e.g. heart disease) and ‘solution’ (e.g. weight loss) is justified. In this section, we identify some instances where public health policy makes reference to responsibility. It will be helpful to have a rough idea of what responsibility, within the philosophical literature, is taken to mean (we will expand on this later).

Responsibility is a way of connecting an agent to some consequence or state of the world—often an action, omission, or set of beliefs. A distinction is commonly drawn between *causal* and *moral* responsibility. Causal responsibility identifies when an actor plays an important causal role in bringing about a particular consequence. Moral responsibility is standardly taken to indicate that the role an agent played in bringing about a consequence means that she is worthy of a particular kind of reaction, such as praise or blame. Merely playing a causal role in bringing about some consequence is generally seen as insufficient for moral responsibility.

Most accounts, going back to Aristotle’s *Nicomachean Ethics* (Aristotle, 2009), assume that for an agent to be morally responsible for an act or omission, she must have had control over whether or not she performed it, and further, she must have been aware of its likely consequences. These are referred to, respectively, as the ‘control condition’ and the ‘epistemic condition’.

In ordinary usage, ‘responsibility’ is normally used with no qualifying term (e.g. ‘causal’ or ‘moral’), but contextual information and non-verbal communication can make it clear whether the kind of responsibility being referred to is strictly causal, or whether it should invoke moral evaluation. For instance, ‘the high winds were responsible for the bridge collapsing’ shouldn’t evoke moral evaluation, since ‘wind’ is not an agent and not subject to this kind of assessment. Thus, ‘responsible’ here must be a causal claim. In contrast, ‘the engineer’s sloppiness was responsible for the bridge collapsing’ does seem to indicate that one would be justified in *blaming* the engineer for her failure to construct a better bridge, and thus points to moral responsibility.

We suggest that in some instances where responsibility is used in health promotion, the kind of responsibility invoked is ambiguous, and is at least open to interpretation as meaning moral responsibility. This is sufficient to motivate the question of whether or not it is appropriate to invoke moral responsibility in the context of public health promotion. For instance, the Department of Health’s *NHS Constitution for England* describes how:

The NHS belongs to all of us. There are things that we can all do for ourselves and for one another to help it work effectively, and to ensure resources are used responsibly… [including recognising] that you can make a significant contribution to your own, and your family’s, good health and wellbeing, and take personal responsibility for it.

NHS England (2015)

Directing individuals to ‘take personal responsibility’ for their (family’s) health is ambiguous. It need not confer an assertion that failure to adopt healthy behaviours (for oneself and one’s family) confers moral criticism, and indeed, NHS England might insist that this is not how they mean ‘take responsibility.’ But, as we will see, given the way in which smoking, drinking heavily and being obese are routinely depicted, it is not a stretch to interpret ‘responsibility’ here as conferring moral responsibility; and for failure to ‘take responsibility’ to be interpreted as a blameworthy failure (Brown, 2018).

Efforts to promote healthier choices are now widespread, incorporating informational and educational interventions, environmental ‘nudges,’ regulation, incentives, and punitive disincentives. The focus on changing individual behaviours in order to tackle chronic disease—particularly the uneven distribution of chronic disease across different social groups—has been described as ‘lifestyle drift’ (Carey *et al.*, 2017). Discussion of lifestyle drift is typically critical of what is perceived to be a focus on downstream influences on health (e.g. calories on a plate), rather than upstream factors (e.g. poverty) which contribute to inequality more broadly and are proposed to be explanatory of the social determinants of health (Marmot *et al.*, 2010). Some of these approaches to health promotion make use of responsibility, implicitly or explicitly, while others sideline the role of responsibility. In the following sections, we discuss how responsibility plays a role in some of these approaches to health promotion.

#### Informational and educational interventions

Informational and educational interventions involve raising awareness of the danger of unhealthy lifestyles and encouraging the avoidance of risk factors by promoting ‘healthy choices.’ For example, the NHS (National Health Service) Choices *Live Well* website provides informational resources relating to at-home fitness workouts, how to reduce dietary sugar, guidelines about alcohol consumption, and tips to quit smoking (NHS, 2017).

In attempting to raise awareness about the harmful effects of unhealthy behaviours, such campaigns might indicate that it is within the individual’s control to avoid those behaviours, and moreover, that she *should* avoid them. Such campaigns are often depicted as ‘empowering’ healthy choices: giving people the information (and thus power) they need in order to adopt healthier lifestyles. For instance, the ‘5 A DAY’ campaign and ‘traffic light’ labelling on food packaging are strategies to encourage healthy dietary behaviours via information and education.^1^ This link between the availability of choice and the creation of responsibility is apparent in policy documents. For instance, a 2010 White Paper produced by the UK Department of Health asserts:

In future, patients and carers will have far more clout and choice in the system… We are also clear that increasing patient choice is not a one-way street. In return for greater choice and control, patients should accept responsibility for the choices they make, concordance with treatment programmes and the implications for their lifestyle.

(Department of Health, 2010: 16)

As such, approaches to health promotion involving information provision and education as means of encouraging healthy choices can also be seen as encouraging responsibility.

#### Nudges, changing environments and regulation

Some approaches to behaviour change manipulate environments to influence behaviour through unconscious pathways, or to explicitly limit or prohibit certain choices. These approaches include ‘nudges,’ such as changing defaults in ways that make healthier behaviours more likely (for example, providing a salad instead of chips as the default accompaniment to a burger) (Thaler and Sunstein, 2008). Different approaches to regulation may also be used in order to make target behaviours less frequent, often by making certain products or activities harder to access or less appealing. This could include a range of strategies, such as coercive regulation restricting access (e.g. preventing the sale of cigarettes to people under the age of 18) or voluntary self-regulation by companies (e.g. to reduce the salt content of processed foods). These approaches do not typically seek to encourage the processes of autonomous deliberation in individuals, but instead make use of non-deliberative tools in order to change behaviour.

One might propose that responsibility is not obviously relevant to nudge-type interventions and regulation, and we wouldn’t disagree. But the absence of a role for responsibility is worth considering. Instead of emphasising the need for individuals to take responsibility for healthy choices, environmental interventions de-emphasise the role of individual choice, and emphasise instead the role of manufacturers, sellers and those shaping the environment where health-related behaviours take place. If responsibility is at all relevant here, it is in relation to those (often group) actors, rather than the individuals whose health is of concern.

#### Penalising unhealthy behaviours

A plausible example of strongly ‘responsibilising’ health policy is through directly penalising unhealthy behaviours, due to the apparent desert basis of such an approach, which assumes that it is appropriate for (some of) the costs created by an individual’s behaviour to be borne by the individual herself.^2^

In practice, few policies (in the UK at least) fall within this category. However, examples could include fines for missed medical appointments, or more controversially, the decision by Hungarian healthcare providers to deny the more expensive form of (analogue) insulin to diabetic patients who failed to stick to their medically recommended diet (Huffington Post, 2012; NY Daily News, 2012). Another plausible example is the decision by a number of clinical commissioning groups in England to delay access to elective surgeries for smokers and obese people unless they quit smoking/lose weight. This has been criticised by the Royal College of Surgeons on the basis that there is no clinical indication for delaying surgery on these grounds, with the suggestion that this amounts to unfair discrimination against marginalised groups in order to save money (Royal College of Surgeons, 2016; Shaw, 2016). Although not presented as a punishment or explicitly justified on desert grounds, since such a policy involves providing a poorer standard of care to certain groups on the basis of their behaviour, it thus might be characterised as penalising those groups relative to non-smokers/non-obese people.

#### Popular portrayals of unhealthy behaviour

While policy rhetoric and intervention design generally stop short of explicitly depicting people as blameworthy for adopting unhealthy behaviours, the popular press tends to show less reserve. Examples of negative stereotyping of those with behavioural risk factors are plentiful in the media:

‘Fat people SHOULD be told that their size is their own fault, experts warns’ [sic] *The Daily Mail* (The Daily Mail, 2016)

‘Obesity is NOT a disability and it’s time fat people started taking responsibility for themselves’ *The Mirror* (The Mirror, 2014)

‘Smoking has become a public declaration of stupidity’ *The Australian* (The Australian, 2015)

‘Katie Hopkins: “Obese people look lazy and are unemployable”’ *The Sun* (The Sun, 2013)

‘Trump’s Budget Director Says Fat, Lazy Americans Don’t Deserve Health Care’ *New York Magazine* (New York Magazine, 2017)

It is worth bearing in mind that, although such depictions cannot be taken as representative of any state’s approach to those adopting unhealthy behaviours, it is a prominent feature of the public discussion surrounding lifestyle-related disease and indicative of how highly moralised this domain of health has become. As one of us has argued elsewhere, the moralisation and stigmatisation of unhealthy behaviour are linked to the portrayal of such behaviours as falling within individual control and responsibility (*Anonymised for review*). It is within this context that explicitly or implicitly responsibilising health promotion operates.

### Components of Responsibility

In order to discuss the legitimacy of state health promotion which encourages people to ‘take responsibility’ for their health, it will be helpful to introduce a little more detail regarding the content of accounts of moral responsibility. Nothing that we say turns on accepting a particular account of moral responsibility, and there are a number of different candidates available with various merits/drawbacks. In the following sections, we will make use of two influential approaches to identifying the conditions for moral responsibility. These come from Watson (2004) and Fischer and Ravizza (1998). While they differ in specifics, these accounts are sufficiently compatible with one another so as to permit drawing insights from both, when discussing the implications of using moral responsibility *in some form* in health promotion. We draw upon Watson’s distinction between different ‘faces’ responsibility to consider whether or not the conditions of responsibility are present in relation to health related behaviour. We then use Fischer and Ravizza’s work in discussing what kinds of reasons need to be present in order for moral responsibility to even get going.

Gary Watson has identified a distinction within moral responsibility between *accountability* and *attributability*, where different responses to an agent are justified, depending on which of these kinds of moral responsibility she bears (Watson, 2004). Watson describes these as two ‘faces’ of responsibility, arguing that attributability is a more basic version of moral responsibility, necessary but not sufficient for accountability. In the case of attributability, an agent’s actions express her agency, and render her subject to ethical assessment. Accountability goes further, and permits that the agent is suitably subject to public remonstration, censure, and the like, for failing to meet social standards of acceptable behaviour [pp. 272–274]. Since accountability carries with it the justification for social practices of blaming, and other similarly disadvantageous responses, the requirements for identifying accountability are more robust than those for identifying attributability. Watson places a high degree of control central to accountability, but requires a lesser degree of control for attributability (a complete absence of control would indicate that a given action is not attributable to an agent). To repeat: both attributability and accountability require that the agent is morally responsible for her actions, but they describe *different kinds* of moral responsibility, and they justify *different responses* on the basis of that responsibility.

Key to Fischer and Ravizza’s canonical account of moral responsibility is the notion of ‘guidance control’ (Fischer and Ravizza, 1998). An agent has guidance control in performing a particular act or omission when she is both ‘moderately reasons-responsive’ and able to take ownership of the action [p. 70]. Fischer and Ravizza thus make reasons central to their understanding of the epistemic and control conditions for responsibility: agents must have the capacity to recognise and respond to moral reasons, as well as access to the practical means necessary to act in accordance with those reasons, if they are to be suitable candidates for moral responsibility. Different factors can limit an agent’s capacity to recognise and respond to moral reasons, such as lacking relevant information, or having diminished cognitive capacities. We will return to the idea of reasons-responsiveness and the role of reasons in evaluating moral claims about agents’ behaviour in Part II.

### Moral Responsibility for Health-Related Behaviour

One approach to considering the justification (or otherwise) of responsibilising health policies is to assess the extent to which agents’ behaviour appears to meet the conditions of moral responsibility implicit in such policies. Policies which treat people as accountable will require that agents have a greater degree of control than policies which only imply attributability. We consider what might be required for accountability and attributability below, and whether it is likely that these conditions are typically met in the context of health-related behaviour.

#### Accountability for health

Policies which hold people accountable for their health can be described as ‘robustly responsibilising’. These could include policies denying liver transplants for alcoholics, prioritising non-obese patients for surgery, or which in some way punish or disadvantage those deemed responsible for their health condition *on the basis* of their role in contributing to the development of that condition. Alternative explanations (and perhaps, justifications) for such policies could be put forward. For example, it could be argued that although the policy appears to hold people accountable, it is in fact aimed only at effectively promoting health. For instance, a defence of many CCGs’ decision to restrict surgery to some groups could be attempted on the basis that those groups have an insufficiently good prognosis (Pillutla *et al.*, 2018). Alternatively, it may be acknowledged that the policy does proceed as if people are accountable, but that this is solely a consequentialist strategy—that holding accountable is merely an effective way of encouraging desired behaviours/distribution of resources, and doesn’t involve any deep claim about a particular individual’s or group’s desert.

Even if the first, ‘prognostic’, explanation is the case, such policies could still be interpreted or experienced as ‘holding people accountable’, with resultant effects of responsibility allocation, blaming and shame. Further, given the use of responsibility language in agenda-setting policy documents quoted above, it seems implausible to suggest that responsibility plays no role in subsequent policies and practice, in cases where there is a clear responsibility-based interpretation of their justification. Given this, such policies can plausibly be considered as involving accountability, insofar as they deny treatment, assistance or support to which people *would otherwise be entitled* (i.e. assuming there are no clear effectiveness-based reasons for denying treatment), on the basis that they have knowingly and avoidably contributed to their poor health. We should consider whether such a basis for denying assistance can be justified.

Empirical research has been used to question the extent to which agents can be considered morally responsible for their lifestyle-related poor health (Brown, 2013). Two strains of research, in particular, have had an important impact on debates about responsibility for unhealthy lifestyles. First, work exploring the social determinants of health has established that poor health outcomes, particularly resulting from chronic disease, are more common among more deprived groups. Extensive research shows that a social gradient exists in health, such that inequalities in power, money, and other resources contribute to a greater risk of suffering from diseases, and reduced (disability-free) life expectancy among those who are worse off (Marmot *et al.*, 2010).

Second, work in health and social psychology suggests that we can model behaviour as operating through two systems: a fast, impulsive and largely unconscious system (I), and a slower, reflective, conscious system (II). Habitual behaviours (including diet, exercise, smoking, etc.) are generally under system I control, and largely responsive to environmental cues, making it difficult to alter them according to reflective preferences (Strack and Deutsch, 2004; Kahneman, 2012).

This research can be taken to show that features of the socio-economic environment, and facts about how human behaviour is ordinarily regulated, partially explain why people often engage in unhealthy behaviour.^3^ At least, it seems reasonable to suggest that an explanation for people’s ‘unhealthy habits’ that assumes people are free to choose between healthy/unhealthy alternatives, and that their behaviour reflects their preferences, is deficient.

The implications of such research for assessing responsibility are not straightforward. However, we can make some rough approximations. If we accept Watson’s analysis that accountability demands high levels of control, then it is likely that at least some people, some of the time will fail to meet such a standard. Indeed, many people who try to lose weight or quit smoking struggle and fail. Even with the best support available, good outcomes may be modest, suggesting that control is limited (Lancaster *et al.*, 2000; Douketis *et al.*, 2005; Franz *et al.*, 2007).

One of us has argued that even those with addictive behaviours, such as drug addicts, can be considered to behave autonomously since evidence suggests that they continue to be reasons responsive (a requirement of responsibility on Fischer and Ravizza’s account) (*Anonymised for peer review*). However, it doesn’t follow from this that people should be held accountable for unhealthy behaviours for three reasons. First, Watson’s accountability requires more control over one’s behaviour than reasons responsiveness alone. Second, even if we accept that reasons responsiveness is sufficient for accountability, the fact that some people are accountable doesn’t mean that everyone is. In practice it will be difficult to discriminate between these two groups, and a policy of holding accountable is likely to inappropriately hold accountable the non-accountable. Third, even if someone is minimally reasons responsive (e.g. if a gun was held to her head and she needed to resist eating cake on pain of death, she would be able to do so) this doesn’t mean that the *actual* reasons she has are able to guide her behaviour. People often have important reasons which do not present as the most salient or powerful at the crucial time point. Strong reasons to lose weight or quit smoking may be overlooked in favour of immediate hedonic pleasure. This does not mean that, all-things-considered, an agent prefers immediate hedonic pleasure to long-term health, but only that different reasons are differently suited to motivating in particular situations.

Such doubts provide a basis for thinking that robustly responsibilising health policies are inappropriate. This would render it inappropriate to deny or withdraw ordinarily provided healthcare on the basis that the recipient is responsible for her poor health, where this relates to health-harming behaviours that are habitual in nature, socially patterned and strongly subject to environmental influences. This is broadly consistent with other criticisms of robustly responsibilising policies (Wikler, 2002; Glantz, 2007; Shaw, 2016; Friesen, 2017).

#### Attributability for health

Let us turn now to moral responsibility as attributability. Insufficient control over ‘lifestyle’ behaviours to justify accountability does not preclude attributability. On Watson’s account, attributability serves an expressive function. By seeing actions as attributable to an agent, we identify those actions as expressing something about her: her values, preferences, concerns, and so on. So long as only attributability (and not accountability) holds, we must stop short of engaging in the public processes of criticism, blame and censure. We may, though, judge an agent well or ill in a more private sense, insofar as her actions are *hers*, and reflect some aspect of her identity.

While agents may have a limited capacity to alter their dietary, exercise or smoking habits, those behaviours with which an individual regularly engages are, in an important sense, her own. Although historical, environmental and psychological factors play a key role in determining the sorts of health-related habits we develop (and struggle to discard), to suggest that such factors alienate an agent from her actions to such an extent that they are no longer attributable to her would seem to require identifying whole swathes of behaviour as non-attributable. Recall, moral responsibility requires the fulfilment of both epistemic and control conditions. The exact requirement of these conditions will vary according to the account of responsibility adopted. On Watson’s account, the control condition is more demanding if the agent is to be considered accountable for her actions, and less demanding if those actions are to be considered merely attributable to her, but she is not accountable. Without providing much more discussion and justification, we cannot specify here exactly what the parameters are for an agent to be considered to have met the control condition for attributability but not accountability. Note that the control condition for attributability could be taken to be very low (i.e. very little control required) such that attributability attaches to all but completely involuntary behaviours.

In the context of health promotion, attributability won’t justify robustly responsibilising policies that blame or punish agents (since these involve public recrimination/censure), but could support more weakly responsibilising health promotion. This could include the use of informational/educational campaigns which instruct agents to make healthy lifestyle changes.

Given that the conditions for attributability could be set very low, and thus be inclusive of much behaviour, this would seem to be supportive of policies which encourage people to ‘take [moral] responsibility’ for their health. We think, however, that there might be a more fundamental problem with identifying attributability here. To see this, return to the idea of guidance control, underpinning Fischer and Ravizza’s account of moral responsibility. Guidance control, recall, is proposed as a necessary condition for moral responsibility, and requires that agents be reasons-responsive in a reliable way.^4^ As Fischer and Ravizza put it:

The kind of responsiveness required for moral responsibility ought to be characterized not merely as a responsiveness to reason, but rather as a responsiveness to a range of reasons that include *moral* reasons.

Fischer and Ravizza (1998: 81)

The use of responsibility in the design and implementation of health promotion interventions, and the tone of popular media discussion of health-related behaviours, is indicative (though, we acknowledge, not conclusive) of the view that people’s health behaviour holds moral relevance: that people have *moral reasons* to pursue healthy lifestyles, and can be criticised for failing to do so. So far, we have argued that the second part of this claim is flawed, since people may lack sufficient control over their health-related behaviour to be held accountable. We will now argue that there is a more fundamental objection to the use of moral responsibility in health promotion: agents often lack moral reasons to adopt healthy behaviours, and thus, moral responsibility cannot even get going. Much of the bioethics literature relating to moral responsibility and health implicitly accepts the assumption that health-related behaviours are ‘moral’ in the relevant sense, and lays out different understandings of moral responsibility from which the case of health is considered (Glannon, 1998; Yoder, 2002; Cappelen and Norheim, 2005; Buyx, 2008; Persson, 2013). Yet if health-related behaviour does not fall within the moral domain, then this discussion may be of less relevance than at first it seems. We will now discuss the *kinds* of reasons agents have to be healthy, and how these may ground responsibility.

## Part II: Prudential Reasons and Responsibility

### Prudential Reasons to Adopt Healthy Behaviours

We have suggested that, at least some of the time, the necessary conditions to consider people accountable for their health will not be present. However, it is plausible that health-related choices are generally attributable to agents, and that agents have normative reasons to adopt healthy behaviours. Here, we argue that these reasons are primarily prudential in nature, rather than moral.

A normative reason is a reason (for an agent) to perform or abstain from performing a certain act. It is a ‘consideration that counts in favour of’ an agent’s acting in a certain way (Scanlon, 1998). Reasons differ in their normative strength, and often weigh alongside other reasons, which might point in the same or opposing directions, and may be weaker or stronger (Parfit, 1984). Having a reason to do something does not oblige one to do it or make it irrational for one not to do it; the reason must be considered alongside all the other reasons for or against the act, which will determine what the agent has all-things-considered reason to do. If a prudential reason to maintain or improve health is strong enough for a particular agent, such that maintaining or improving health is what the agent has all-things-considered reason to do, then it will be rational for the agent to act on that reason.

Philosophers often identify different species of normative reasons. One variety, *moral* reasons, can be grounded in the wellbeing of others—the interests others have in their wellbeing gives the agent moral reasons to promote and protect the wellbeing of others. Another variety, *prudential* reasons, are principally grounded by the agent’s own wellbeing—they are self-regarding: the interest the agent has in her wellbeing gives her prudential reasons to promote and protect it.^5^

We suggest that the normative reasons people have to maintain or improve their health are predominantly prudential in nature. This does not preclude the operation of moral reasons alongside prudential reasons, and we indicate what basis there might be for moral reasons in the context of adopting healthy behaviours in the penultimate section. Even if health is not itself constitutive of wellbeing, good health has a significant instrumental role in enabling agents to accrue the experiences, events or objects that are. This will be the case on three of the main approaches to understanding well-being: mental state/hedonistic theories; desire fulfilment theories; and objective list theories, as well as composite theories that incorporate elements of each (Parfit, 1984; Savulescu, 2006). Being so, agents have reasons to maintain their health to a level sufficient to maintain or increase their wellbeing. Further, sufficient health is necessary for continued life, which is a prerequisite for (although does not guarantee) high levels of wellbeing. It is important to stress that, although health is often instrumental for wellbeing, engaging in behaviours that are detrimental to health can also sometimes increase an agent’s wellbeing, depending on their particular interests and values.

### Prudential Responsibility

Given that agents have an interest in their wellbeing, and that a sufficient level of health is instrumental for wellbeing on any account, agents consequently have a responsibility *to themselves* to respond to instrumental reasons to maintain it to a sufficient level. This, we argue, grounds a plausible concept of self-regarding prudential responsibility. The notion of prudential responsibility for maintaining health is principally prospective, providing guidance for future behaviour. To the extent that health is instrumental for wellbeing, agents have prudential reasons to maintain or increase it. Unlike moral responsibility, however, failure to fulfil one’s prudential responsibilities (by not acting upon one’s prudential reasons, for instance, to quit smoking) will not (generally) render one subject to external moral criticism.

Prudential responsibility may justify self-directed criticism for failure to act in ways consistent with one’s prudential reasons. For instance, one may reproach oneself for overeating when one is trying to lose weight. Prudential responsibility may also hold in the absence of the capacity/control conditions described above: one may have prudential reasons for quitting smoking, and a prudential responsibility to attempt to do so, even in the absence of the required skills, such as self-restraint, to be successful.

The strength of direction arising from an agent’s prudential responsibility to maintain her health, and the specific acts it encourages or discourages, will vary from agent to agent. Assuming that we accept some subjective dimension to wellbeing, the strength and content of an agent’s prudential reasons will depend in part on what she finds pleasurable and painful (and to what extent), and on the preference ranking of what she desires. External third parties such as the state cannot therefore assume that every agent has the same prudential reasons, of the same strength, to maintain the same level of health.

Despite non-uniformity in prudential reasons and responsibility, on most understandings of well-being agents should, prudentially, maintain a sufficient level of health. Although this will vary between agents, a level of health that is free from significant disease is likely to be prudentially valuable to most agents—call this ‘threshold health’. Given that threshold health is necessary for most prudential goods for most agents, it is permissible for states to appeal to agents’ prudential responsibility in the context of health promotion, to facilitate agents to act in line with their prudential reasons to maintain their threshold health, either by making agents more aware of their prudential reasons, providing information needed to understand how to act on those reasons, as well as the practical resources required to do so.

An objection to the use of prudence in health promotion might come from a recent article in which Stephen John discusses ‘prudentialism’ in alcohol policy (John, 2018). John describes prudentialism as an approach to policy which seeks to target ‘irresponsible’ drinkers while not interfering with ‘responsible’ drinkers. John argues that, although there are reasons for thinking prudentialism gets something right, it also endorses categories of people (in this case, particular groups of irresponsible/responsible drinkers) which incorporate troubling social norms.

We think this is a reasonable concern, highlighted by the fact that the targets for criticism of unhealthy behaviour (drinking, smoking, factors contributing to obesity) are overrepresented in lower socio-economic groups. Other factors which risk health (skiing, marathon running, foreign travel) are less likely to be labelled as ‘irresponsible’ on the basis that they create avoidable costs. The categories of responsible/irresponsible thus appear not only to track health risk, but also class and wealth.

We do not, however, think our proposal of adopting an approach to health promotion which focuses on facilitating prudential responsibility necessitates endorsing such troubling categories of responsible/irresponsible behaviour. Our discussion is aimed at reinforcing the importance of individual values and circumstances in determining what prudential reasons someone has, and the extent to which adopting healthy behaviour will be prudentially responsible for a particular individual, given her circumstances and preferences. Since health promotion is largely a blunt instrument, we cannot tailor interventions to people’s specific needs, but instead aim to promote threshold health, likely to contribute to the majority of agents’ interests. We thus cannot guarantee that efforts to develop interventions which facilitate people’s prudential responsibility will not end up tracking the troubling social categories that concern John. We think that John’s account provides a useful test for health promoting interventions: a need to check whether the groups targeted by health promoters are in fact proxies for groups typically stigmatised for other reasons, or whether a focus on them is justified on the basis of valuable gains in health and well-being.

### Moral Reasons and Duties to Maintain Health

Let us return to the question of whether, in addition to prudential reasons to maintain health, agents also have moral reasons to do so. Impartialist accounts (such as utilitarianism and Kantianism) claim that *all* reasons for action are moral reasons. In contrast, egoistic accounts stipulate that reasons for action are always essentially self-interested. Other accounts permit prudential and moral reasons to diverge.

On a utilitarian moral theory, prudential reasons to maintain or improve one’s health may at the same time be moral reasons, if one’s failure to act in line with these reasons will predictably lead to one’s life containing less net utility. Thus, according to utilitarianism, agents have moral as well as prudential reasons to maintain their health, where this is instrumental to their wellbeing, and where there is not some alternative act that would increase wellbeing more, at some cost to their health. On a Kantian account, the morally required action (which must represent one’s strongest reason) will be determined by the categorical imperative to act rationally and in accordance with universalizable maxims, and thus leaves no room for divergence between reasons for action deriving from morality and those from one’s self-interest.

These impartialist accounts of normative reasons may have further implications for responsibility, denying that what we call prudential responsibility can be sensibly distinguished from the more established concept of moral responsibility. Those adhering to utilitarianism or Kantianism are thus unlikely to accept our analysis of prudential responsibility, and we offer no direct response here. However, accepting that prudential and moral reasons come apart allows us to make sense of ordinary understandings of everyday examples. Consider an individual who fails to develop a successful career due to a lack of focus and application. She experiences less well-being over her lifetime, and her failure to flourish might be seen as regrettable by concerned third parties. However, we do not typically think her failure should be considered a *moral* failure, that provides others with an appropriate justification to criticise her on moral grounds, far less punish her or interfere with her freedom to choose how to act in this domain. We propose that the health behaviour case is similar: that agents’ prudential reasons to maintain their health provide a normative ‘ought’ such that they *should* (often) adopt healthy behaviours, but that this does not constitute a moral responsibility to do so. This does not preclude the possibility for moral reasons to act alongside prudential reasons, such that an agent could have both moral *and* prudential reasons to adopt/refrain from a particular behaviour.

While we argue that only prudential, and not moral responsibility is applicable (in the main) to health related behaviour, one might reject this claim and yet accept some of the implications of our position. Our practical concern is with whether the existence of a moral responsibility to adopt healthy behaviours can be used by the state in designing health policy, and for this it is not essential to accept that prudential reasons and moral reasons are separable, but only that the reasons people have to adopt healthy behaviours do not justify the kinds of actions by the state as would be justified if individuals were failing to fulfil their moral obligations. Put another way, if individuals are behaving in ways that ignore their moral responsibilities, there will be initial grounds for thinking that the state could be permitted to step in to take action to ensure that people *do* fulfil their moral responsibilities (such as removing their access to ordinarily available services, or using punishments or penalties to enforce morally obligatory behaviour). We propose that the kinds of reasons people have to adopt healthy behaviours are not moral reasons which ground moral obligations, and so do not provide even an initial indication that state enforcement of behaviours that track those reasons would be justified on these grounds. This sets aside whether state enforcement of healthy behaviours could be justified on alternative grounds.

This question of enforceability may ultimately be more important, since the identification of distinct prudential and moral reasons will not always be straightforward. Following Henry Sidgwick, a number of moral philosophers endorse a so-called ‘dualism of practical reason’. (Sidgwick, 1874; Smith, 2009; Crisp, 1996) This highlights the potential for conflict between what morality requires and what is in one’s own self-interest: either can create reasons that serve to motivate and justify action in different circumstances. On theories that allow for this genuine dualism of practical reason, prudential reasons might overlap with or be *supplemented by* moral reasons. We consider now the prospect for an independent basis of duties (based on moral reasons) to adopt healthy behaviours. We briefly consider three candidates for such duty-claims below: solidarity; special relationships; and self-regarding concerns.

#### Solidarity

It might be argued that all citizens have solidaristic obligations to avoid being free-riders on the healthcare system. Alternatively, we might consider individuals as having general civic duties which require them to contribute to the shared goal of public health promotion, via the personal adoption of healthy behaviours. The exact demands of solidarity are unclear, but could involve not taking more from the system than one contributes; or one’s ‘fair share’; or than is needed for a minimally decent life, for instance.^6^ Within a solidaristic system, there may be different strengths of obligation, some of which are strong direct moral obligations, some of which may be weaker, supererogatory and indirect. Obligations could extend beyond maintaining one’s health, to other ways in which one contributes to society, for instance, by developing one’s talents or creating important works of art.

Solidaristic systems rely on people acting cooperatively and to some degree, altruistically, if they are to work efficiently. There is a danger of ‘moral hazard’ when the costs of risky behaviour are spread among a large number of people. In healthcare, we might risk our health by smoking, eating cake and never exercising, knowing that the costs of future treatment will be covered by the system, and spread among all taxpayers or others’ insurance premiums.

Although appealing, the empirical basis of solidarity-based duties to adopt healthy behaviours is questionable. Economists suggest that smokers contribute more to the economy than non-smokers, due to taxes on cigarettes and the nature of the illnesses they typically suffer (Wilkinson, 1999). Evidence for other ‘lifestyle’ behaviours is less clear, but, as Wilkinson argues, claims that those adopting risky behaviours exploit socialised welfare systems would logically extend to a whole range of other behaviours, which begins to look implausibly demanding.

#### Special relationships

While it does not seem plausible that we have general obligations to others to preserve our health, it might be the case that we have particular obligations that we owe to those with whom we share special relationships. This could include loved ones, dependents, employers and employees: those whose wellbeing is significantly affected by our behaviour. If one sabotages one’s health through sustained exposure to extremely harmful behaviours, rendering one incapable of caring for one’s child or showing up for work, it looks like both the child and employer have a legitimate grievance. This suggests that there exist instrumental duties to maintain one’s health, at least to the minimal level required in order to fulfil one’s primary duties to those with whom one sits in a special relationship. This might establish moral reasons for individuals to adopt minimally healthy behaviours. Where such moral reasons exist, they will only create obligations to adopt healthy behaviours to the extent that this is achievable for the particular agent (given people’s sometimes limited control over their behaviour, as discussed above).

#### Self-regarding obligations

Finally, a broadly Kantian line of reasoning would specify that one has duties towards oneself, on the basis of preserving one’s humanity or avoiding self-contradiction and irrationality (Kant, 1785/1998). Such duties could require one to adopt behaviours likely to preserve one’s health, providing distinct reasons from the instrumental prudential reasons to pursue wellbeing already discussed.

While we do not deny the (potential) existence of moral reasons indicated by duties towards those with whom we sit in special relationships or towards ourselves, the scope of such reasons for grounding moral responsibility and, further, justifying approaches to health promotion by the state which require that agents ‘take responsibility’ for their health (and may make healthcare provision dependent on adopting healthy behaviours) is limited. First, the demands of these duties are likely to require lower levels of health and wellbeing than those established by the prudential reasons discussed above. Rendering oneself incapacitated to the extent that one can no longer meet the basic standards of care for a child or maintain employment will involve losses beyond the threshold health required to meet prudential responsibilities.^7^

Second, duties based on the special relationships between agents will not be broadly applicable, since although most agents will hold some such duties, there will be agents who sit in no duty-creating special relationships with other people. Kantian duties towards oneself will, however, apply to all agents. Whether one accepts that such duties exist depends on how persuasive one finds such Kantian arguments (we acknowledge that if one accepts such an account, one might grant general moral reasons to adopt healthy behaviours).

Finally, it is not clear that, even where agents have duty-grounding moral reasons to be healthy, this creates the conditions for moral responsibility that can legitimately be enforced by the state. Since our concern here is with state efforts to promote health through responsibilising policy, this is key. For instance, even were we to accept that one owes it to one’s partner to refrain from smoking, this does not provide a basis for the *state* to intervene to force one to fulfil those obligations and quit smoking, for example, by implementing policies which restrict one’s access to future healthcare on the basis of one’s smoking status.

### Concluding Remarks and Practical Implications

We have argued that the encouragement of people to ‘take responsibility’ for their health, and the depiction of people as morally responsible for health harms that result from behavioural risk factors, rests on contestable assumptions about people’s moral responsibility for health-related behaviour. We propose that the incorporation of responsibility into health promotion strategies should be restricted to a narrow form of prudential responsibility. We do not, here, offer an all-things-considered argument against the use of responsibilisation in health promotion. Rather, we suggest the limited ways in which people appear morally responsible for their health-related behaviour provide a compelling case for adopting a presumption against wide and unrestricted responsibilisation. Such a position opposes ‘robustly responsibilising’ policies which restrict treatment or punish individuals whose lifestyles have contributed to their disease. In this concluding section, we offer some tentative suggestions at to the implications of this approach for health promotion policy.

In so far as we propose individuals have a prudential responsibility to adopt healthy behaviours, states, in promoting the interests of citizens, should facilitate (but not enforce) the fulfilment of this responsibility. This is broadly in line with many current policies of health promotion and advocated elsewhere (Coggon *et al.*, 2016), including policies which aim to provide people with information about the impact of ‘lifestyle’ behaviours on their health, highlighting agents’ potential roles in maintaining their health, and facilitating opportunities to act in line with prudential reasons. However, the important implication of our argument is that public health initiatives invoking moral responsibility will not be justified. Our concept of prudential responsibility therefore assists with the task of determining which instances of communication engage with individuals’ prudential reasons vs. apparent moral reasons, and provides the justification for those instances that engage with the former type of reasons. Further, health promotion initiatives rooted in concerns for prudence must be cognisant of the fact that unhealthy behaviours will be differentially detrimental to individuals’ wellbeing, and differently amenable to change. What counts as prudent behaviour will ultimately be determined by features of the individual: what she finds pleasurable, what preferences she holds, the sorts of activities she values, and her hopes and plans for the future. While some accounts of wellbeing emphasise its objective components, most assume sensitivity to individual preferences and circumstances are important.

**Figure 1.**
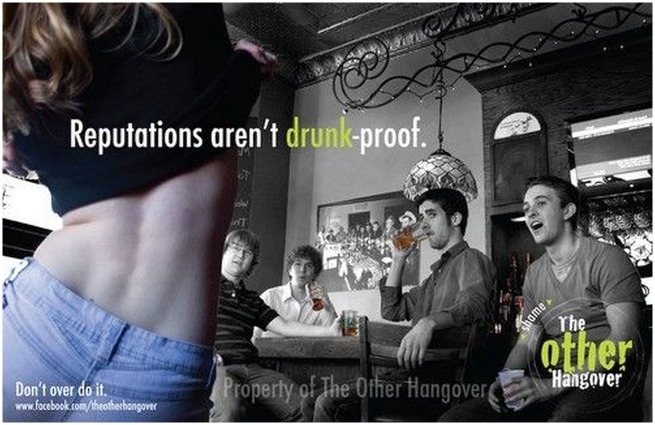
Reputations aren’t drunk-proof, 2011 (image courtesy of the Other Hangover campaign, University of Minnesota).

So, permissible public health campaigns must accommodate the facts that (i) people will differ in the extent to which they have reasons to avoid unhealthy behavior, and (ii) the reasons individuals have are prudential, not moral, and failure to act in line with prudential reasons is not blameworthy. For example, campaigns concerning alcohol consumption can permissibly appeal to the ways in which alcohol might reduce short- or long-term wellbeing (perhaps by affecting sleep, mood, mental clarity or relationships) and provide strategies, support lines or groups, and information about the effects of alcohol on the body. Such campaigns should not assume that abstinence is prudentially optimal for all people, nor that everyone will have all or any of the possible prudential reasons to cut down. Language must also avoid any implications that drinking is morally objectionable/indicates moral failure in the individual. We can see the difference this makes in the following examples:

Figure 1 appeals to reasons rooted in the apparent shamefulness of ‘loss-of-control’ behaviour that alcohol might facilitate, and the reduced social standing of those whose behaviour changes when drinking alcohol. The image might be interpreted in different ways, and could be objectionable on grounds distinct from responsibilisation (e.g. misogyny). We think it is also an example of moral responsibilisation: while it will be the case that prudential reasons to refrain from drinking large qualities will be present for most people, the emphasis on reputation and social evaluation is moralising, reinforcing the stigma that attaches to people (particularly women) who drink heavily. It is true that individuals’ wellbeing will often be affected by how people view them, and thus in one sense, the poster flags individuals’ prudential reasons for avoiding heavy alcohol consumption. But by exploiting and reinforcing the source of these interests (i.e. the harm that results from stigma and shame) posters such as this are foreseeably moralising and, we argue, impermissible.

Another example comes from an anti-smoking campaign run by the British Heart Foundation. It features a poster with the following text:

SYMPTOMS

Bad Breath

Stained Teeth

Clothes Stink

Bad Skin

Always Broke

CONCLUSION

SMOKER

This clearly invokes negative evaluation of the smoker, emphasising effects of smoking that are presented as garnering social disapproval. The effects that are predominantly aesthetic will be especially subject to variation in the extent to which individuals have prudential reasons to avoid them. Highlighting bad skin and stained teeth, for example, does not engage with prudential reasons unless the individual places high value on a polished appearance. The suggestion that these are ‘symptoms’ is particularly stigmatising, suggesting that being a smoker is itself a disease.

**Figure 2.**
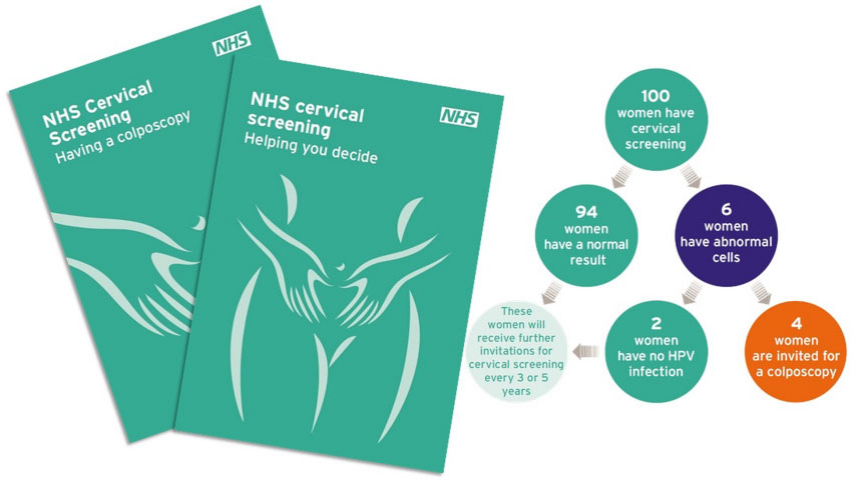
NHS cervical screening information leaflet, 2016 (image courtesy of Public Health England).

In contrast, a leaflet produce by the NHS to inform women about cervical screening (Figure 2) is careful and explicit in describing the harms and benefits of screening. It provides information about population average effects including the risks of false positive tests, and advises people that it is their choice whether or not to attend screening, based on whether they think it is likely, on the whole, to be of benefit to them. We propose that such messaging is consistent with supporting prudential responsibility.

We have further summarised how moral responsibility- and prudential responsibility-based health promotion compare in Table 1. Often, health promotion strategies assuming moral and prudential forms of responsibility will have overlapping implications. For instance, both will justify providing key health-related information and education, and permit non-responsibilising policies (such as making use of ‘choice architecture’) be used in addition/as an alternative to responsibilising policies. The table is intended to be illustrative rather than comprehensive, and there will be many further considerations affecting whether or not a given policy is justified.

**Table 1.** Illustrating the policy implications of moral and prudential responsibility-based approaches to health promotion

Behaviour / health domain	Moral Responsibility-based approach	Prudential Responsibility-based approach
Smoking (health promotion to encourage cessation)	Treatment for smoking-related ill health de-prioritised; smokers required to pay (some proportion of) the costs of treatment for smoking-related ill health; information / education emphasising individuals’ moral obligations to quit smoking; stigmatising, moralising and shaming campaigns criticising smoking behaviour.	Treatment for smoking-related ill health provided as for any other health condition; information / education to indicate how smoking may negatively affect health and other interests; efforts to avoid stigmatisation, moralisation or shaming which harms smokers’ interests; development of non-harmful alternatives (e.g. vaping technologies) to maintain value derived from smoking.
Alcohol (efforts to reduce high levels of consumption)	Treatment for alcohol-related ill health de-prioritised (e.g. liver transplants preferentially directed towards those with non-alcohol-related disease); those harmed by alcohol consumption required to pay (some proportion of) the costs of their treatment; stigmatising and shaming campaigns highlighting ‘bad behaviour’ of excessive drinking.	No treatment discrimination between alcohol-related and non-alcohol-related ill health; information provided on potential health impact of alcohol consumption; enable drinking in ways likely to enhance prudential interests but discourage drinking in ways likely to harm them (e.g. targeting ‘binge drinking’); directly combat stigmatisation and shaming of those drinking to excess.
Diet and physical activity (to combat overweight and obesity)	Restrict treatment for people with avoidable ill health on the basis of desert; provide information regarding recommended diets and physical activity, plus the means of securing these behaviours (healthy foods available in supermarkets, access to spaces to exercise, etc.); use contact with healthcare professionals as opportunities to challenge people’s lifestyles; explicitly criticise people for failing to take opportunities to maintain a healthy weight through diet and physical activity; permit stigmatising, moralising and shaming campaigns to make overweight and obesity socially unacceptable.	Treatment for overweight/obesity-related ill health in line with non-weight related disease; acknowledge that dietary / exercise behaviours have different value for different people, and that people might reasonably prefer less healthful behaviours; challenge negative stereotyping; provide guidance as to likely ways of improving quality of life via diet and physical activity in ways likely to be helpful for the majority, and facilitate access to the necessary components of those behaviours.

This article is intended as exploratory, and space constraints limit discussion of potential objections. One such objection might be: why should the state refrain from effective use of moral responsibilisation merely because of uncertainty about people’s *genuine* moral responsibility? We recognise the importance of considering effectiveness in making all-things-considered decisions about appropriate health promotion strategies. There might be instances where policies that are overtly moralising are very successful at changing behaviour and improving health. Holding people morally responsible for their health might form a convenient social fiction that allows governments to implement effective health promotion strategies. We think such ‘government house utilitarianism' should be resisted. Knowingly enacting policies based on inconsistent, erroneous reasoning is not a behaviour of states that we should accept. While we would not rule out the possibility of exceptions, where there is an extraordinarily high pay off, it is unlikely that responsibilising health promotion is such an exception, since most such policies are modestly, if at all, effective (Puhl and Heuer, 2010).

A further concern may come from criticisms of lifestyle drift, mentioned earlier. Recall, those critical of lifestyle drift argue that health promotion has become overly focused on individualistic explanations of chronic disease, distracting from upstream causes such as poverty (which contributes to the overrepresentation of chronic disease in more deprived populations). One might argue that prudential responsibility is likely to still permit such individualising in public health. We recognise that there could be political motivations to emphasise individualistic approaches which our discussion is not designed to counter. While we, too, are sceptical of the move towards individualism, and take seriously those who argue change must be directed at much larger, complex social structures, this is not the problem we seek to tackle here. It is, we think, perfectly compatible with prudential responsibility that one also holds that tackling upstream causes of poor health is both ultimately more effective and more equitable than seeking to promote health by changing individual behaviours. This does not, however, mean that it would be inappropriate to also provide people with information that is relevant to their personal projects, where ‘relevant’ here means that it is within their power to act differently on the basis of that information.

We have not discussed autonomy as a basis for considering the legitimacy of responsibilising approaches to health promotion. The promotion and preservation of autonomy could provide distinct reasons for (or against!) adopting healthy behaviours, which do not track either people’s prudential interests or their moral obligations. We do not have space to address how considerations of autonomy should be integrated into an account of responsibilising health promotion, but consider it an interesting and important topic for future philosophical work.

## Footnotes

1. 5 A Day is an NHS campaign recommending people eat at least five portions of fruit and vegetables per day; traffic light labelling uses colours to indicate nutrient content of foods.

2. It might be argued that such penalising approaches could be purely consequentialist, and that they need not make any assumptions about desert in explaining their use. Yet purely consequentialist justifications of punishment are unlikely to be acceptable to any but committed utilitarians, and so we assume this is an unlikely basis of justification for such policies.

3. There is much more that could be said here about other areas of relevant research, considering evolutionarily adaptive behaviours, the role of marketing and promotion of business interests, political influence, and so on.

4. Watson is somewhat critical of Fischer and Ravizza’s account, concerning the details of how agents must respond and / or react to reasons for them to be considered capable of guidance control. We consider what reasons are present in the context of adopting healthy behaviours, rather than how agents respond to those reasons, and so we set Watson’s concerns aside.

5. There are other types of normative reasons, grounded by objects or domains of value, such as legal norms or aesthetic values.

6. For further discussion of solidarity-based welfare systems see Glannon (1998).

7. With exceptions, such as preference-based accounts of wellbeing where individuals derive pleasure from extremely risky behaviours. In such cases, one’s moral obligations towards others might require higher levels of health than would be needed to fulfil one’s prudential responsibilities. We assume such cases are rare.
